# COVID-19 Variability Within European Countries Sourced From ECDC Data. Is Variability Explained by Specific Country Policies?

**DOI:** 10.3389/fpubh.2021.737133

**Published:** 2022-01-18

**Authors:** Alberto Ruano-Ravina, Esther López-Vizcaíno, Cristina Candal-Pedreira, María Isolina Santiago-Pérez, Mónica Pérez-Ríos

**Affiliations:** ^1^Área de Medicina Preventiva y Salud Pública, Universidad de Santiago de Compostela, Santiago de Compostela, Spain; ^2^CIBER de Epidemiología y Salud Pública, CIBERESP, Madrid, Spain; ^3^Health Research Institute of Santiago de Compostela (IDIS), Santiago de Compostela, Spain; ^4^Servicio de Difusión e Información, Instituto Galego de Estadística, Santiago de Compostela, Spain; ^5^Servicio de Epidemiología, Dirección General de Salud Pública, Consellería de Sanidade, Santiago de Compostela, Spain

**Keywords:** COVID-19, variability, epidemiology, European Centre for Disease Prevention and Control (ECDC), health policy

## Abstract

**Background:**

Europe has had a large variability in COVID-19 incidence between and within countries, particularly after June 2020. We aim to assess the variability between European countries and regions located in a given country.

**Methods:**

We used ECDC information including countries having 7 regions or more. The metric used to assess the regional variability within a country was the intercuartilic range in a weekly basis for 32 weeks between June 29^th^ 2020 and February 1^st^ 2021. We also calculated each country's overall variability across the 32 weeks using the distances from the regional curves of the 14-day incidence rates to the corresponding national curve, using the L^2^ metric for functional data. We afterwards standardised this metric to a scale from 0 to 100 points. We repeated the calculations excluding island regions.

**Results:**

The variability between and within countries was large. Slovenia, Spain and Portugal have the greatest variability. Spain and Slovenia held also the top three places for the greatest number of weeks (Spain for 19 weeks and Slovenia for 10) with the highest variability. For variability among the incidence curves across the 32-week period, Slovenia, Portugal and Spain ranked first in functional variability, when all the regions were analysed but also when the island regions were excluded.

**Conclusions:**

These differences might be due to how countries tackled the epidemiological situation. The persistent variability in COVID-19 incidence between regions of a given country suggests that governmental action may have an important role in applying epidemiological control measures.

## Introduction

Europe ranks high among the areas hardest hit by the COVID-19 pandemic. By early March 2021, the continent had recorded 21,765,152 cases of the disease and 531,896 COVID-19 deaths since the pandemic began (1). During the period commonly known as the first wave, most countries were taken by surprise at the lack of preparedness for a health crisis of such unprecedented proportions in times of peace in Europe. This crisis manifested itself in many ways, ranging from the scarcity of diagnostic tests, the absence of personal protective equipment for healthcare staff and nursing homes workers, and the saturation of hospitals due to the high numbers of cases, to the lack of knowledge about how to break the chain of infection (2). All of this led to a generalised lockdown being implemented in most countries across Europe. After application of these stringent public health measures, most of these countries reported a significant decline in incidence by the end of June 2020. Even so, from October 2020 onwards, incidence across Europe began climbing again, leading to the so-called second and third waves (3). These post-October increases in incidence have not been uniform across the European Union (EU), whether in terms of the peaks reached or the points in time when these occurred. Thus, whereas France and Spain registered peaks in incidence at the end of October and beginning of November 2020, not only was the French peak twice as large as the Spanish peak, but France, unlike Spain, experienced no subsequent peak. Furthermore, throughout almost the entire period, incidence in Germany was lower than that seen in France and Spain. These differences point to an effect attributable to the different ways in which the pandemic was managed in each country.

In addition, incidence was observed to vary widely among the regions making up the respective EU countries (4–6). Some authors have sought to explain this variability by proposing a series of causes, classifiable as demographic in nature or related to public health measures. Chief among the demographic factors are household structure (with intergenerational cohabitants or not), population density, residential occupant density, lifestyles or customs in the individual countries, and the frequency of use of meeting places such as bars, cafes and restaurants. Similarly, the mean age of the population also plays a role, as does population mobility. Even climatic aspects which may necessarily imply more life being spent indoors could be brought under the demographic umbrella. On the other hand, the implementation of public health measures, such as confinement, lockdown of non-essential economic activity and schools, mandatory wearing of face masks, implementation of curfews or closure of hospitality establishments, or even more or less stringent restrictions at Christmas time across countries are all believed to have an impact on COVID-19 incidence (7–9).

While the above-mentioned demographic factors can, to all intents and purposes, be regarded as uniform within a given country, this does not apply to public health measures introduced to control the pandemic in cases, such as Spain, where healthcare action is highly decentralised. In a scenario of this kind, measures implemented by the different regional authorities around the country can play a key role in achieving greater or lesser control of COVID-19 incidence (2, 10). All this may have contributed to the wide difference in COVID-19 incidence observed, both between European countries and between the different regions of such countries.

There is little evidence on the variability of COVID-19 incidence across Europe and the reasons that may account for this phenomenon. The formal approach to its analysis is complex: some authors have focused on variability in mortality and the lethality rate (6, 11, 12); others have analysed how COVID-19 incidence behaves at a national level (5, 13); and while one study has analysed variability in incidence across the European continent, it does not take into account variability between the regions of a single country (4). Nonetheless, there are relatively simple methodologies which allow for a single metric to be applied to both inter-country variability and intra-country variability by region, such as the interquartile range of variation in incidence between regions. This variation, applied to different points in time (after the first wave) when the necessary evidence and material capacity to manage the pandemic were obtained, makes it possible to evaluate and compare the respective performances of European countries. This would, in turn, enable one to ascertain whether greater or lesser variability in COVID-19 incidence between regions occurs sporadically, or in contrast, whether there is sustained variability over time that might indicate management failures by the relevant health authority.

We feel that the study of the dynamics of COVID-19 transmission in European countries could prove crucial in guiding decision-making concerning public health measures. Accordingly, this study aimed to compare COVID-19 incidence among EU countries and between the different regions of these same countries, across the period 29 June 2020 (week 27 in 2020) to 7 February 2021 (week 5 in 2021).

## Methods

### Data-Sources

The main data-source used was the European Centre for Disease Prevention and Control (ECDC). On 10 February 2021, we downloaded the weekly 14-day COVID-19 case notification rates per 100,000 population for the 27 EU countries, both nationally and regionally, until week 5 in 2021, from the ECDC website (https://www.ecdc.europa.eu/en/covid-19/data). Population data at 1 January 2020 were sourced from EUROSTAT (http://appsso.eurostat.ec.europa.eu/nui/show.do?dataset=demo_r_pjangrp3).

By way of an inclusion criterion, countries were required to have seven or more regions, in order to ensure that the calculation of variability would be based on a sufficient number of regions. This number of regions was chosen for two main reasons: the first one was to exclude countries with only one region or very few regions (Malta, Cyprus, Luxemburg, with only one region, and Belgium with three regions), and also to exclude countries with very low incidence during the period analysed (<1,500 accumulated COVID cases/100.000 inhabitants) and therefore with a very low expected variability. This was the case of Denmark and Latvia. In addition, Norway was eliminated, since population data on the regions furnished by the ECDC were not available in EUROSTAT, with the result that data on a total of 20 countries with 333 regions were analysed.

To perform a sensitivity analysis of the data, the study was replicated with the island regions excluded. In general, and due to their geographical characteristics, these regions have been observed to have a lower disease incidence and could therefore introduce an element of additional variability in countries which include such territories. There was a total of 18 island regions.

### Statistical Analysis

To ensure that countries were in comparable situations vis-à-vis the COVID-19 epidemic, we used data on the 14-day case notification rates as from week 27 in 2020, once the first wave had passed (29 June 2020).

The data supplied by the ECDC at a regional level had negative values in the 14-day incidence rates for some weeks and regions. To resolve this situation, the negative values were eliminated, and these rates were then imputed from the remaining data using spline interpolations (14).

For each country and week considered, the median, the first and third quartile, and the interquartile range of the 14-day incidence rates were calculated and plotted on charts showing country trends. The aim was to identify those countries which displayed the greatest degree of variability in their rates.

Furthermore, for each week of the period analysed, countries were ranked in descending order according to their interquartile ranges, and those in the top three positions were identified so as to calculate the number of weeks during which each country was in the upper ranges of variability with respect to the others.

To quantify each country's overall variability across the 32 weeks of the period analysed, we calculated the distances from the regional curves of the 14-day incidence rates to the corresponding national curve, using the L^2^ metric for functional data for this purpose (15, 16). The following expression was used:


$$\begin{array}{l} \text{D}\left({\text{C}}_{\text{ij}}\left(\text{t}\right),{\text{C}}_{\text{j}}\left(\text{t}\right)\right)=\sqrt[{2}]{\int {\left({\text{C}}_{\text{ij}}\left(\text{t}\right)-{\text{C}}_{\text{j}}\left(\text{t}\right)\right)}^{2}} \end{array}$$

where C_ij_(t) is the curve of region i in country j and C_j_(t) the curve of country j (t indicates the week).

The regional distances of each country were then used to calculate an average, weighted for the population of the regions, which would be an equivalent of standard deviation, *via* the following expression:


$$\begin{array}{l} {\text{d}}_{\text{j}}=\frac{\sum_{\text{i }=\;1}^{{\text{n}}_{\text{j}}}{\text{p}}_{\text{ij}}\text{ D}\left({\text{C}}_{\text{ij}}\left(\text{t}\right),{\text{C}}_{\text{j}}\left(\text{t}\right)\right)}{\sum_{\text{i }=\;1}^{{\text{n}}_{\text{j}}}{\text{p}}_{\text{ij}}} \end{array}$$

where n_j_ is the number of regions in country j and p_ij_ is the population of region i in country j. The result, d_j_, is a measure of the functional variability of country j.

Lastly, to facilitate interpretation of the measure d_j_, the values of all the countries were standardised on a scale scored from 0 to 100, by means of the following transformation:


$$\begin{array}{l} {\text{I}}_{\text{j}}=\frac{{\text{d}}_{\text{j}}-\min\left({\text{d}}_{\text{j}}\right)}{\max\left(\text{dj}\right)-\;\min\left({\text{d}}_{\text{j}}\right)}\;*\;100 \end{array}$$

where d_j_ is the weighted distance or functional variability of country j.

Hence, the country with the smallest distance or variability has a value I_j_ = 0, and the country with the greatest distance has a value I_j_ = 100. The remaining countries have a scale value of 0 to 100, which indicates their relative position, in terms of variability, between the country with lowest variability (country 0) and the country with highest variability (country 100).

All analyses were performed using Stata v14·2 and R (fda.usc and ggplot2 packages).

## Results

By 31 January 2021, ~33.5 million COVID-19 cases had been diagnosed in Europe, amounting to a cumulative incidence of almost 4,000 cases per 100,000 population. In the 20 EU countries included in this analysis, cumulative incidence was 4,364.6 cases per 100,000 population (18.5 million cases). A breakdown by country indicated that the minimum incidence was observed in Finland and the maximum incidence in Czechia, with 869 and 9,741 cases per 100,000 population respectively. Table 1 shows the data of the countries included, with total cumulative incidence and 14-day incidence in the last week of analysis (week 5 in 2021). It will be seen that in this week, the highest incidence rate (1,190 cases per 100,000 population in Portugal) was approximately 12 times higher than the lowest (98 cases per 100,000 population in Finland).

**Table 1:** Description of countries included.

**Country**	**Population (k)**	**COVID-19 cases**		**Number of regions**		**Region population (k)**	
		**14-day rate per 100k on week 5-2021**	**Cumulative incidence per 100k**	**Total**	**Islands**	**Median**	**Range**
Austria	8,859	224.8	4757.5	9	0	755	293–1,897
Bulgaria	7,000	127.4	3196.2	27	0	167	85–668
Croatia	4,076	157.5	5776.7	21	0	139	45–806
Czechia	10,650	914.6	9741.1	14	0	609	295–1,369
Finland	5,518	97.7	869.3	19	1	181	30–1,671
France	67,013	423.0	4979.7	18	6	3,324	269–12,245
Germany	83,019	176.9	2756.7	16	0	3,271	683–17,933
Greece	10,725	111.6	1528.7	13	4	574	204–3,742
Hungary	9,773	176.4	3864.4	20	0	365	189–1,752
Ireland	4,904	326.5	4150.9	8	0	473	304–1,388
Italy	60,360	281.5	4368.4	21	2	1,640	126–10,061
Lithuania	2,794	353.3	6693.9	10	0	177	94–811
Netherlands	17,282	317.9	5825.2	12	0	1,136	383–3,709
Poland	37,973	196.4	4088.9	16	0	2,086	946–4,489
Portugal	10,277	1190.1	7448.1	7	2	705	243–3,573
Romania	19,414	175.5	3845.8	42	0	398	194–1,830
Slovakia	5,450	496.4	4845.2	8	0	668	564–825
Slovenia	2,081	762.2	8361.7	12	0	116	53–549
Spain	46,937	843.1	6368.3	19	2	1,488	85–8,427
Sweden	10,230	394.1	5812.8	21	1	287	59–2,344

*Cumulative cases per million population until week 5/2021, number of regions per country, and minimum, median and maximum population*.

The 20 countries are divided into 333 regions, with wide variability among the countries, both in the number of regions and in their population sizes, as can be seen from Table 1. Thus, the countries with the most highly populated regions are France, Germany and Poland, with a median of over 2 million inhabitants. The 18 island regions belong to 7 countries, and of the 5 that belong to France, 4 are overseas island territories.

**Figure 1:**
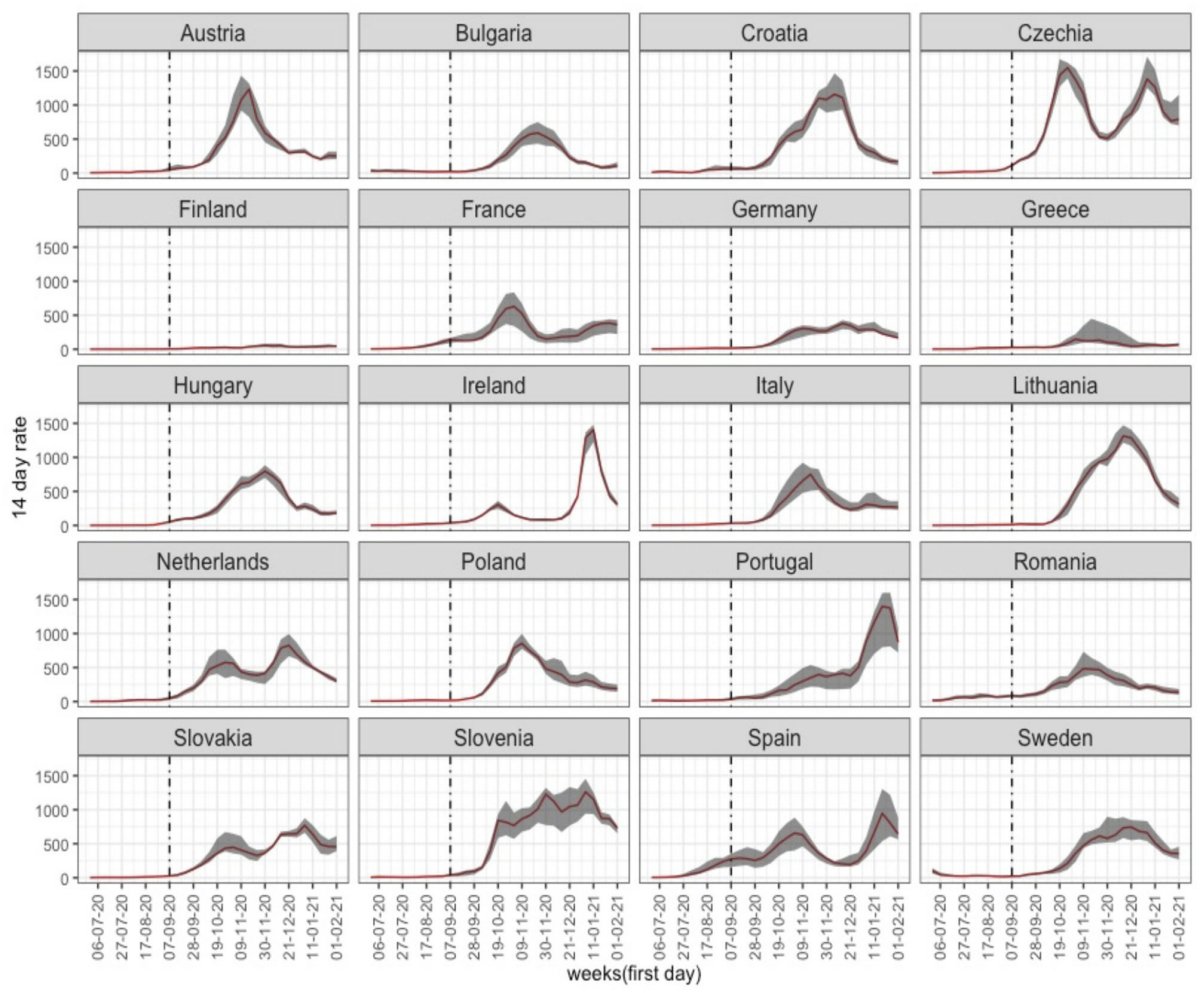
Fourteen-day COVID-19 incidence rates in 20 EU countries from week 27/2020 to week 4/2021. Median regional rates in each country (red line) and interquartile range (grey band).

Figure 1 depicts the weekly trend in the median 14-day incidence rate per country, and its interquartile range from week 27 in 2020. A different pandemic pattern is observable among countries, in incidence levels as well as in variability between regions. The greatest variability would appear to be found in Slovenia, Spain and Portugal. It is also apparent that until 7 September (see vertical line), incidence was very low in all the countries analysed except for Spain, where incidence began to rise in July.

**Figure 2:**
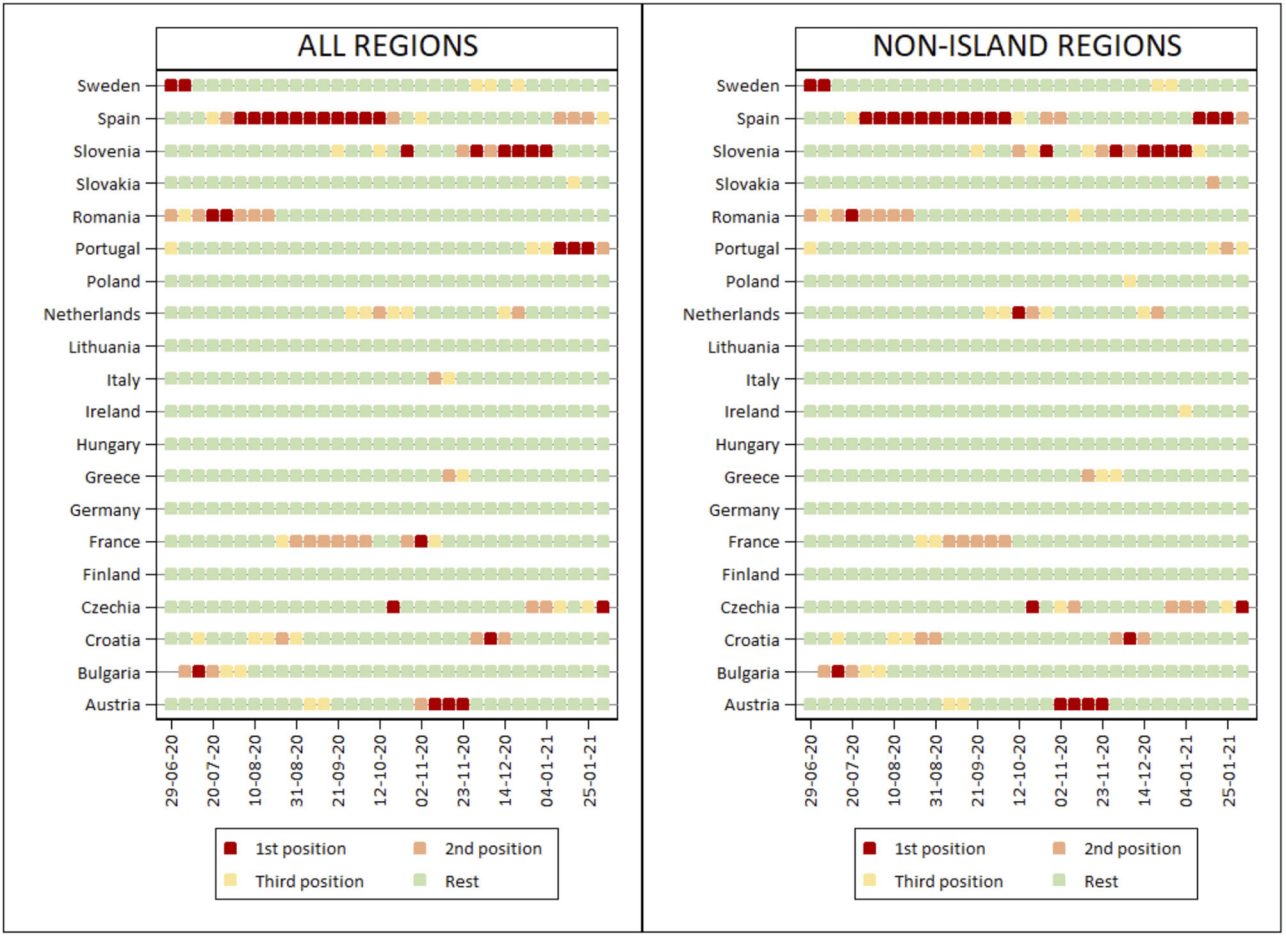
Ranking held by countries in each week of the period analysed according to their interquartile range, in descending order of value.

Figure 2 and Table 2 show the countries with the greatest interquartile range for each of the weeks analysed, indicating which countries ranked first, second and third in this range. Spain and Slovenia were the countries which held the top three places for the greatest number of weeks (Spain for 19 weeks and Slovenia for 10). This classification of countries remains largely unchanged if the island regions are eliminated from the analysis (19 and 13 weeks respectively). It should be stressed that there is a single country, namely Spain, with two peaks of maximum variability, corresponding to points of low incidence (summer) and high incidence (end of autumn) at a European level.

**Table 2:** Number of weeks during which each country held first, or second-third place according to their interquartile range, in descending order of value.

**Country**	**Number of weeks with highest interquartile range**			
	**All regions**		**Non-island regions**	
	**First place**	**Second/third place**	**First place**	**Second/third place**
Austria	3	3	4	2
Bulgaria	1	4	1	4
Croatia	1	7	1	7
Czechia	2	4	2	6
Finland	0	0	0	0
France	1	9	0	7
Germany	0	0	0	0
Greece	0	2	0	3
Hungary	0	0	0	0
Ireland	0	0	0	1
Italy	0	2	0	0
Lithuania	0	0	0	0
The Netherlands	0	7	1	6
Poland	0	0	0	1
Portugal	3	4	0	4
Romania	2	6	1	8
Slovakia	0	1	0	1
Slovenia	6	4	6	7
Spain	11	8	14	5
Sweden	2	3	2	2

If variability among the incidence curves is analysed across the 32-week period, Slovenia, Portugal and Spain are the three countries that rank first in functional variability, not only when all the regions are analysed but also when the island regions are excluded (Table 3). It will be noted, moreover, that the measure of variability of these three countries is far higher than that of the remaining countries, though variability does decrease slightly in Spain and Portugal when the island regions are excluded.

**Table 3:** Functional variability of each country, weighted by the population of the regions, using the L^2^ metric.

**Country**	**Functional variability using all regions**		**Functional variability using non-island regions**	
	**Original**	**0–100 scaled**	**Original**	**0–100 scaled**
Slovenia	1061.3	100	1061.3	100
Portugal	1061.0	100	981.8	91
Spain	1003.6	93	955.4	88
Czechia	920.1	84	920.1	84
Croatia	820.9	72	820.9	72
Sweden	761.2	65	760.6	65
France	749.3	64	714.5	60
Italy	733.4	62	718.0	60
Romania	642.8	51	642.8	51
Austria	627.4	49	627.4	49
Slovakia	608.8	47	608.8	47
Lithuania	555.5	41	555.5	41
Poland	534.1	39	534.1	39
Netherlands	529.9	38	529.9	38
Greece	489.3	33	487.9	33
Bulgaria	467.7	31	467.7	31
Hungary	381.7	21	381.7	21
Ireland	372.7	20	372.7	20
Germany	307.5	12	307.5	12
Finland	202.7	0	202.6	0

*Countries shown in descending order of variability*.

## Discussion

Our results highlight the fact that there are three countries which display a far greater degree of regional variability than any other European country. In the case of Spain, it ranked among the top three in variability for 60% of the period analysed (19 of 32 weeks), followed at some distance by Slovenia. These results may imply differences caused by aspects related to management of the pandemic at a national level, as well as factors linked to lifestyle and/or demographic or household structure.

Due to the lack of activation of internationally homogeneous response mechanisms, each country acted independently, seeking to find measures that would work in its territory (17). Consequently, despite the fact that certain public health measures, such as home confinement of the population, were implemented in all European countries, there was a wide divergence in the timing of their introduction by the respective governments. In Italy, confinement began on 22 February, and in Spain confinement was not implemented until 14 March (17). Added to this are the criticisms levelled at governments, on the one hand, for announcing stringent measures too late, and on the other, for the perceived economic and social damage flowing from these measures (17). In much the same way as European countries had failed to implement the measures uniformly, the easing of such measures was likewise introduced irregularly, as to both pace and intensity, thereby contributing to wider variability in incidence after the first wave. In this respect, there were important differences between the incidence thresholds set by governments to classify population risk. Hence, in Spain, extreme risk was defined as more than 250 cases per 100,000 population in the previous 14 days (18), though exceeding this risk did not entail the imposition of any additional mandatory measure by the government. Indeed, it was left to the discretion of the regions to apply (or not to apply) such measures, thus making for highly variable health policy action throughout the nation. Such potential variability will necessarily be greater, the higher the number of regions capable of taking decisions in any given country.

That said, however, public health measures are not the only factor to be borne in mind when trying to account for variability in incidence across Europe. Socio-demographic aspects have been observed to have a great impact on virus transmission patterns and, by extension, on incidence in European countries (19). Higher COVID-19 incidence has been associated with higher population density and a higher percentage of inhabitants in urban areas of the country (19–21). It is possible that countries with a region having one or more major cities may have recorded a higher incidence, as these cities are much more densely populated. Major cities have different transport systems (subway, buses, trams, etc), that allow for the movement of many people, facilitating the movement of asymptomatic persons from one place to another. This fact would potentially drag the incidence of that region upwards. Furthermore, the shorter the distance between the home unit and a major city, such as the capital of a country, the higher the COVID-19 incidence caused by greater population mobility. In general, the outskirts of European cities are home to large populations, often with lower income levels, a factor that has also been associated with a higher incidence of COVID-19 cases in New York (22).

In addition to the above factors, population structure seems to play an important role. The uneven distribution of COVID-19 cases may be due to the mean age of the population, in that older populations are associated with a higher incidence of symptomatic cases of COVID-19 (19). In young populations there may be a greater number of real but asymptomatic cases, implying under-detection in this population stratum. An ageing population, in turn, gives rise to a higher percentage of institutionalised persons in nursing and old age homes, where the disease has been seen to have had a great impact (23). Then again, it should also be borne in mind that in countries situated in southern Europe, such as Spain, Portugal and Italy, it is commonplace for several generations to live together in a single home, entailing a risk of intergenerational transmission, which is proportionally greater in smaller living spaces. As a result, intergenerational contacts occur more frequently, accelerating virus transmission in an older and, therefore, more vulnerable population (24). This occurs less frequently in countries situated in the north of Europe. One study conducted in New York indicates that there is a correlation between incidence of infection and home size, which is in turn related to the number of people living there. It is estimated that, for each additional person who resides in a home, the number of COVID-19 infections rises by 46.4% (22).

Account should also be taken of the fact that culture and social behaviour differ according to the country and, even, the region, and can have an influence on COVID-19 incidence. The density of leisure venues, such as bars, cafes and restaurants, in southern European countries is usually higher. It should be noted that populations in the south of the continent tend to spend more time outside the home, in contrast to inhabitants of northern countries where, for instance, there is a widespread tendency to stay home.

The results of this study suggest that island regions play a key role in COVID-19 variability within European countries. In the case of France, with 18 regions, 5 of which are overseas territories, the island regions have a great influence on the variability of COVID-19 incidence. In other countries, such as Spain or Italy, with two island regions each and a large number of mainland regions, their relative influence is less pronounced. At all events, islands are generally observed to have a lower incidence than do mainland territories. This may be due to the fact that accessibility to islands is limited, which makes for less mobility of persons and greater control of travellers, and therefore reduces incidence (13).

In Spain, wide variability occurred, not only in months during which COVID-19 incidence was low across the whole of Europe (July, August, September), but also when incidence was generally high (November, December, January), heading the European rankings in both cases. One might surmise that the above patterns would show a certain similarity between Spain, Italy and Portugal, situated in the south of Europe, with similar populations, cultures and household structures. Yet these countries have not witnessed the extraordinary variability in incidence observed in Spain, so that factors associated with suboptimal management of the pandemic might well explain these differences.

This study has a number of limitations. The fact that it is an ecological study which compares variability between countries at different points in time means that the results could be due to any variable or variables that we have not considered. Examples of these worth mentioning include residential occupancy rates, mean age of the population, population awareness in the different countries, educational or risk communication measures, and even per capita income.

This study also has a number of strengths. Firstly, the data source used is homogeneous and independent, and makes it possible to work with an outcome variable that is uniformly measured in all countries. We also consider it an advantage that the design used excluded the first months of the pandemic, when ignorance as regards how to tackle it, coupled with the lack of resources (including the lack of availability of diagnostic tests), may have generated important differences between countries. A further advantage is that we have used the L^2^ metric for functional data, which is recognised as a strong metric to analyse and compare the incidence curves of the different regions. This statistic has been scarcely used to analyse COVID-19 variability across territories. Lastly, the fact of having measured incidence at the same time in all countries allows for an accurate temporal comparison of incidence at each point in time.

It can be concluded that there has been a considerable degree of variability in COVID-19 incidence, both among and within European countries. While these differences could be accounted for by phenomena of differential transmission among countries due to socio-demographic factors, they could also be due to the way in which governments tackled the epidemiological situation, once a relative degree of epidemiological control had been achieved at the beginning of summer 2020. The observation of persistent variability in COVID-19 incidence between regions of a given country suggests that governmental action may have been lax in applying epidemiological control measures in some European countries, such as Spain, Slovenia and Portugal.

## Data Availability Statement

Publicly available datasets were analyzed in this study. This data can be found at: European Centre for Disease Prevention and Control, https://www.ecdc.europa.eu/en/covid-19/data and Eurostat, http://appsso.eurostat.ec.europa.eu/nui/show.do?dataset=demo_r_pjangrp3.

## Author Contributions

AR-R and MP-R had the original idea of the research and conceptualised the methodology. CC-P drafted the first version of the manuscript. MS-P and EL-V performed the statistical analysis. All authors take public responsibility of the manuscript content and have approved the final version.

## Conflict of Interest

The authors declare that the research was conducted in the absence of any commercial or financial relationships that could be construed as a potential conflict of interest.

## Publisher's Note

All claims expressed in this article are solely those of the authors and do not necessarily represent those of their affiliated organizations, or those of the publisher, the editors and the reviewers. Any product that may be evaluated in this article, or claim that may be made by its manufacturer, is not guaranteed or endorsed by the publisher.
